# Activated astrocytes drive the accumulation of apolipoprotein E at the brain tumor edge

**DOI:** 10.1007/s10014-025-00511-5

**Published:** 2025-07-31

**Authors:** Ting‐Yi Chien, Chi‐Shiun Chiang

**Affiliations:** Department of Biomedical Engineering and Environmental Sciences, National Tsing Hua University, Hsinchu, 300044 Taiwan

**Keywords:** Astrocytes, Apolipoprotein E, Spatial analysis, Tumor microenvironment, Macrophage

## Abstract

While tumor-associated macrophages (TAMs) have been extensively studied, the role of tumor-associated astrocytes (TAAs) in glioma progression is less explored. Astrocytes are crucial in maintaining lipid homeostasis by synthesizing cholesterol and apolipoprotein E (APOE) in the brain. However, the contribution of astrocytes in supporting the metabolic needs of tumor cells within the tumor microenvironment (TME) is still poorly understood. This study aims to investigate how astrocytes contribute to the unique brain TME by examining the spatial distribution of APOE and its correlation with glial cells. This study examined the spatial distribution of APOE in gliomas with two murine brain tumor models: ALTS1C1 and GL261. To validate astrocyte APOE secretion, in situ hybridization (ISH) for APOE mRNA and immunofluorescence (IF) staining for GFAP were performed. Immunofluorescence (IF) staining showed that APOE was accumulated at the tumor edge. ISH analysis confirmed that activated astrocytes were the primary cells responsible for the increased APOE in this region. Flow cytometry and IF staining demonstrated that TAMs were also associated with increased APOE expression in the tumor core. This study provides the first evidence that astrocytes at the tumor edge are activated and upregulated for APOE secretion. These brain tumor edge-associated astrocytes are responsible for the accumulation of APOE in this region and create a unique metabolic environment, which may contribute to brain tumor invasion and resistance to therapy.

**Supplementary Information:**

The online version contains supplementary material available at 10.1007/s10014-025-00511-5.

## Introduction

An important characteristic of the brain tumor microenvironment (TME) is the specialized cell types found exclusively in the brain, such as microglia, astrocytes, oligodendrocytes, and neurons. Astrocytes are the brain’s dominant glial cells, accounting for approximately half of the brain’s cells. These cells are essential for supporting the unique function of the nervous system [1]. For example, astrocytes are responsible for synthesizing cholesterol in the brain, which is then transported to other cells with apolipoprotein E (APOE), a protein also synthesized by astrocytes in the CNS (central nervous system) [2–4]. Glial fibrillary acidic protein (GFAP) is a well-characterized marker of astrocytes and is commonly used to identify activated astrocytes in pathological conditions, including tumors. Unlike tumor-associated macrophages (TAMs), the roles of tumor-associated astrocytes (TAAs) have long been overlooked [5]. Previous studies have shown that astrocytes may drive the progression of brain tumors [6–8]. Earlier studies in our lab have also found a correlation between carbonic anhydrase IX (CAIX) expression and GFAP + astrocytes at the tumor edge, indicating the alteration in astrocyte metabolism during glioma invasion [9].

Due to the high proliferation rate of glioma cells, dysregulation of lipid homeostasis in brain tumors has been observed. Previous studies have shown that GBM cells rely on exogenous cholesterol for survival, as demonstrated using several GBM cell lines and an orthotopic mouse brain tumor model [10]. One study analyzed patient GBM specimens and revealed that cholesterol synthesis enzymes were upregulated in the tumor core compared to the margin area, suggesting that tumor cells in the core rely more on endogenous synthesis, while those at the margin preferentially take up cholesterol from the microenvironment [11]. Another study reported higher cholesterol levels in the tumor interstitial fluid compared to the brain interstitial fluid based on data from human, rat, and mouse brain tumor models. It was found that APOE accumulated at the GBM tumor tissue margin, with levels higher than in adjacent tissues, both in the mouse GBM model and in patient tissue chips [12]. While most studies focus on targeting the dysregulated cholesterol metabolism of GBM cells, few have investigated the contribution of stromal cells, including astrocytes, to this distinct cholesterol metabolic TME. Furthermore, despite extensive examination of APOE in the context of Alzheimer’s disease progression, few studies have explored its role in the brain TME.

Although many treatment methods have been investigated for glioma, the spatial and temporal heterogeneity of both the tumor and stromal cells makes treatment more challenging [13]. One study utilized single-cell RNA sequencing (sc-RNA-seq) analysis to examine bulk and margin glioma tissue. The results showed that bulk and margin tumor cells exhibit distinct functions, which are predominantly controlled by the extrinsic microenvironment rather than intrinsic mutations [14]. Another study identified a distinct microglia subset at the tumor–stroma interface, which displayed a pro-inflammatory and chemotactic phenotype associated with peripheral monocyte recruitment [15]. Due to the heterogeneous cell composition in the microenvironment, spatially resolved TME is needed for a comprehensive understanding of the potential role of each component in the TME.

This study aims to provide in vivo evidence on how astrocytes may contribute to brain TME. Given that cholesterol secretion involves both de novo synthesis and apolipoprotein-mediated delivery, this study investigates the spatial distribution of APOE and its correlation with glial cells in the brain TME.

## Materials and methods

### Cell line

Murine astrocytoma cell line, ALTS1C1 (T8239, Applied Biological Materials, Richmond, Canada, or BCRC60582, Hsinchu, Taiwan), was previously established in our lab [16]. GL261, a murine glioma cell line, was kindly provided by Dr. Newcomb’s lab at New York University Medical Center. ALTS1C1-GFP cell line was created by lentiviral infection of GFP-expressing vector into the ALTS1C1 cells. ALTS1C1, ALTS1C1-GFP, and GL261 were cultured in Dulbecco’s Modified Eagle’s Medium (DMEM; Gibco, 12100-046, NY, USA) with 10% Fetal bovine serum (FBS; Gibco, 16000-044) and 1% Penicillin–Streptomycin (Gibco, 15140-122). Cells were passaged every 2–3 days and maintained in a humidified incubator at 37 °C with 5% CO_2_. For cell passaging, cells were first washed with 1X PBS (Biological Industries, 02-023-5A, Israel) and dissociated by 0.5% Trypsin–EDTA (Gibco, 15400-054) at 37 °C for 5 min.

### Animal

Six to eight-week-old C57BL/6J male mice were purchased from the National Laboratory Animal Center of Taiwan. All experimental procedures were approved by the Institutional Animals Care and Use Committee (IACUC approval No. 107042) of National Tsing Hua University, Taiwan. All surgeries were performed under anesthesia conditions, and efforts were made to minimize the mice’s suffering. 75% Zoletil 50 (50 mg/mL) (Virbac, France) and 25% Rompun 20 (Bayer, Korea) mixture was used for mouse surgery. Mice were anesthetized by intraperitoneal injection of 2 μL/g Zoletil–Rompun mixture.

### Intracranial injection

Tumor cells were counted and resuspended in DMEM. The cell suspension was kept on ice during the surgery. Animals were anesthetized with a Zoletil–Rompun mixture. The fur of the skull was removed, and the animals were fixed on the stereotactic frame (David Kopf Instrument). A midline scalp incision was created, and a burr hole was made at 1 mm posterior and 2 mm lateral (right) from the bregma by a rotary drill. 2 μL tumor cells (1 × 10^5^ cells/2 μL) were injected into the hole at 2.5 mm depth. The cell suspension was delivered through a microinjection needle at 1 μL/min for 2 min. After injection, the needle was left in the brain for 2 min and then slowly removed from the brain. The burr hole was covered with bone wax (ETHICON, W810, Belgium), and the incision was sutured (UNIK SURGICAL SUTURES MFG, ST174, 75 cm, 30″, New Taipei City, Taiwan). The mice were killed on day 18 after tumor injection.

### Tissue collection for histological analysis

When killed, mice were anesthetized with a Zoletil–Rompun solution. Then, intracardiac perfusion was performed with cold PBS (8 mL, 2 min) followed by cold 4% paraformaldehyde (PFA) (Sigma, 16,005, Germany) (8 mL, 10 min) to fix the tissue. The brain was removed and soaked in 10 mL PFA at 4 °C 18 h for post-fixation. After post-fixation, the brain tissue was transferred to 25 mL 10% sucrose (Sigma, S0389, USA) at 4 °C 2 h and then to 25 mL 20% sucrose at 4 °C 18 h to dehydrate the sample. After dehydration, the tissue was transferred to 5 mL Optimal cutting compound (OCT; Leica, 14,020,108,926, United Kingdom) at 4 °C 2 h to acclimate the OCT compound. After acclimation, the tissue was transferred to a cryomold filled with OCT and left to balance at room temperature (RT) for 30 min. The cryomold was then covered with lab tape to avoid isopentane contamination. The tissue was frozen by isopentane (ACROS Organics, 126,470,010, USA), cooled by liquid nitrogen for 3 min, and then stored at − 80 °C. Frozen tissue was first thawed at − 20 °C 1 h before cryosection. The tissue was sectioned into 10 μm slices by the cryostat (Leica, CM1850, IL, USA) at − 20 °C and adhered to the saline slide (MUTO PURE CHEMICALS, 511,614, Japan). The slide was then stored at − 80 °C.

### Histological analysis—Filipin III staining

The slide was fixed with 1.2% PFA at RT for 10 min and washed 3 times with PBS for 2 min. The tissue was then surrounded by a hydrophobic barrier pen (VECTOR LABORATORY, H-4000, CA, USA). The slide was incubated with 100 µL Filipin III staining solution (250 μg/mL Filipin III in PBS) (Filipin III: Sigma, SAE0087, USA) at RT for 1.5 h and washed 3 times with PBS for 2 min. 100 µL Propidium iodide solution (1 μg/mL Propidium iodide solution in PBS) (Propidium iodide: Sigma, P4170, USA) was added to label the nuclei at RT for 5 min. Following Propidium iodide staining, the slide was washed 3 times with PBS for 2 min and let dry. Finally, 10 μL mounting medium (VECTOR LABORATORY, H-1000-10) was dropped on each tissue slice, and the slide was covered with a coverslip. The image was viewed by a fluorescence microscope (ZEISS, Axioskop 40 CFL, Gottingen, Germany) and captured by the camera (ZEISS, Axiocam 503 color).

### Histological analysis—hematoxylin and eosin (H&E) staining

The slide was fixed with 95% methanol (Merck, 33213-2.5L, Germany) at − 20 °C for 10 min and washed twice with PBS for 5 min. The slide was dipped in Hematoxylin solution (Surgipath, 110,619, Leica Biosystems, IL, USA) for 1 min at RT and rinsed with water. The slide was then dipped in 0.25% ammonium (J.T.Baker, 9721-03, USA) for 10 s and rinsed with water. After air-drying, the tissue was dipped in Eosin solution (Surgipath, 82,219, Leica Biosystems) for 20 s at RT and rinsed with water. The slide was dipped twice in 95% ethanol (Merck, 32221-2.5L, Germany) and air dried. Finally, 10 μL mounting medium (VECTOR LABORATORY, H-5000) was dropped on each tissue slice, and the slide was covered with a coverslip. The image was viewed by a microscope (ZEISS, Axioskop 40 CFL, Gottingen, Germany) and captured by the camera (ZEISS, Axiocam 503 color).

### Histological analysis—immunofluorescence (IF) staining

The slide was fixed with 95% methanol at − 20 °C for 10 min and washed twice with PBS for 5 min. The tissue was then surrounded by a hydrophobic barrier pen. The slide was treated with protease at 40 °C for 8 min and then washed 3 times with PBS for 1 min. To block the non-specific binding, the slide was incubated with a blocking buffer (5% FBS and 0.1% TWEEN 20 (Merck, P1379, USA) in PBS) for 60 min at RT. After blocking, the slide was incubated with the 200 μL primary antibodies diluted in blocking buffer at 4 °C overnight (12–16 h). Following the primary antibody staining, the slide was washed twice with washing buffer (0.1% TWEEN 20 in PBS) for 5 min. A secondary antibody diluted in a blocking buffer was then added to label the primary antibodies. The slide was incubated with 200 μL secondary antibodies at RT for 1 h and was washed twice with washing buffer for 5 min. The slide was then incubated with 200 μL Hoechst solution (1.25 μg/mL Hoechst 33342 in PBS) (Hoechst 33342: Thermo Fisher, 1871965, USA) to label cell nuclei at RT for 5 min. Following Hoechst staining, the slide was washed twice with PBS and let dry. Finally, a 10 μL mounting medium was dropped on each tissue slice, and the slide was covered with a coverslip. The image was viewed and captured by confocal microscopy (ZEISS, LSM780). Antibodies used and the dilution ratio are as follows: CD68 (Bio-Rad, MCA1957GA, USA; 1:200), GFAP (Abcam, ab7260, USA; 1:400), APOE (Sigma, 178479, USA; 1:1000).

#### Histological analysis—in situ hybridization staining

The slide was removed from − 80 °C and let air dry at RT for 2 h. The slide was fixed with 4% PFA at 4 °C for 20 min and washed twice with PBS for 1 min. The tissue was then surrounded by a hydrophobic barrier pen. In situ hybridization staining was performed with ViewRNA ISH Fluorescence Tissue Assay kit (Thermo Fisher, QVT0600C) according to the manufacturer’s instructions. The slide was treated with 100 μL protease at 40 °C for 10 min and then washed 3 times with PBS for 1 min. The slide was re-fixed with 4% PFA at RT for 5 min and then washed 3 times with PBS for 1 min. The slide was then incubated with a 100 μL Apoe target probe (Thermo Fisher, VX-06, assay ID: VB1-10244-VT) at 40 °C for 2 h and then washed 3 times with wash buffer for 2 min. Following target probe hybridization, the slide was incubated with 100 μL pre-amplifier solution at 40 °C for 30 min and then washed 3 times with wash buffer for 2 min. After pre-amplifier hybridization, the slide was incubated with 100 μL amplifier solution at 40 °C for 30 min and then washed 3 times with wash buffer for 2 min. After amplifier hybridization, the slide was incubated with 100 μL label probe solution at 40 °C for 30 min and then washed 2 times with wash buffer for 2 min. Finally, 10 μL Prolong antifade mountant with DAPI (Invitrogen, P36931, USA) was dropped on a tissue slice and the slide was covered with a coverslip. For ISH combined with IF staining, after label probe hybridization and washing, the slide was blocked with 100 μL blocking buffer (4% FBS, 1% goat serum, and 0.1% TWEEN 20 in PBS) at RT for 1 h. The slide was then incubated with primary antibody diluted in the blocking buffer overnight at 4 °C (12–16 h). Following the primary antibody staining, the slide was washed twice with washing buffer (0.1% TWEEN 20 in PBS) for 5 min. A secondary antibody diluted in a blocking buffer was then added to label the primary antibodies. The slide was incubated with 100 μL of secondary antibodies at RT for 1 h and was washed twice with PBS for 5 min. Finally, 10 μL Prolong antifade mountant with DAPI was dropped on a tissue slice, and the slide was covered with a coverslip. The image was viewed and captured by confocal microscopy.

### Brain tissue dissociation for flow cytometry analysis

Mice were anesthetized with a Zoletil–Rompun solution. Intracardiac perfusion with PBS (15 mL) was performed to eliminate blood contamination. The brain was removed from the skull following the removal of the cerebellum and olfactory bulb and then dissected into three parts: the left hemisphere, the right hemisphere, and the tumor. The tumor tissue was removed from the brain and placed in a 6 cm dish prefilled with 4 mL cold PBS, while the brain tissue was transferred into a 50 mL tube prefilled with 2 mL cold DMEM and kept on ice until the next step. For the brain tissue dissociation, the brain tissue with 2 mL DMEM was transferred into the pre-cooled Tenbroeck Grinder (DWK life science, 885000-0015, Mainz, Germany). The tissue was homogenized by gently rotating the pestle while moving the pestle up and down 20 times. After Tenbroeck homogenization, an additional 3 mL DMEM was added into the grinder to a final volume of 5 mL. The sample was then transferred into a 50 mL tube and then homogenized with a syringe (5 mL) and needle (21-gauge) 10 times. The homogenate was then filtered through a 70 μm cell strainer and centrifuged for 5 min at 400 rcf at room RT. After centrifugation, the pellet was resuspended with 1 mL 1X RBC lysis buffer (Thermo Scientific, 00-4300-54, CA, USA) and kept on ice for 5 min. 2 mL PBS was then added to stop the reaction, and the sample was centrifuged for 5 min at 400 rcf at RT. The whole homogenization process was kept on ice to reduce cell death. Percoll solution (GE Healthcare, 17-0891-01, Uppsala, Sweden) was used to remove myelin. A stock solution of isotonic Percoll (SIP) was prepared by mixing 9 mL Percoll solution and 1 mL 10X HBSS (Gibco, 14,185). The cell pellet was resuspended in 4 mL 37% SIP (3.7 mL SIP + 5.3 mL 1 × HBSS) and centrifuged for 20 min at 1200 rcf at RT without brake. After centrifugation, the myelin at the top of the gradient was removed by pipette aspiration. The cell pellet at the bottom of the centrifuge tube was collected.

### Tumor tissue dissociation for flow cytometry analysis

For the tumor tissue dissociation, the tissue was dissected into small pieces with a surgical blade. The tissue was transferred into a 15 mL tube and centrifuged for 5 min at 400 rcf at RT. After centrifugation, tissue was digested by 4 mL diluted ACCUMAX™ solution (STEMCELL TECHNOLOGY, 07921, USA; diluted ACCUMAX™ solution: 1 mL ACCUMAX™ + 4 mL 1X PBS) with gentle shaking for 60 min at RT. The homogenate was then filtered through a 70 μm cell strainer and centrifuged for 5 min at 400 rcf at RT. After centrifugation, the pellet was resuspended with 1 mL 1X RBC lysis buffer and kept on ice for 5 min. 2 mL PBS was then added to stop the reaction, and the sample was centrifuged for 5 min 400 rcf at RT.

### Flow cytometry analysis

To block the Fc receptor, cell pellets were resuspended with blocking buffer and kept on ice for 15 min (blocking buffer: 0.2% FcR blocking reagent (BD, 553,142, USA) and 1% goat serum (Thermo Scientific, 16,210,064) in 1X PBS). To label the surface antigens (CD45, CD11b), 100 μL of cell suspension was added into 100 μL staining buffer containing antibodies (staining buffer: 1% goat serum in 1X PBS) and kept on ice for 30 min. After incubation, 1 mL staining buffer was added to wash out the antibodies, and the cells were centrifuged for 5 min at 400 rcf at RT. Antibodies used to label surface antigens are as follows: CD45 (BD, 552,848; 1:200), CD11b (BD, 550,993; 1:200). Subsequently, cells were fixed and permeabilized with BD Cytofix/Cytoperm Fixation/Permeabilization Kit (BD, 554,714) to label the intracellular antigen (APOE). Cell pellets were first vortexed and resuspended with 200 μL fixation/permeabilization solution for 20 min on ice. After fixation, 1 mL Perm/Wash Buffer was added to wash out the fixation buffer, and cells were centrifuged for 5 min at 1000 rcf at RT. Subsequently, cell pellets were resuspended with 200 μL Perm/Wash Buffer containing antibodies and incubated on ice for 30 min. After incubation, 1 mL Perm/Wash Buffer was added to wash out the antibodies, and the cells were centrifuged for 5 min at 1000 rcf at RT. After centrifugation, cell pellets were resuspended with PBS and stored at 4 °C in the dark until flow cytometry analysis. The antibody used is as follows: APOE (NOVUS, NB110-60531PE, USA; 1:200). Flow cytometry analysis was performed using BD FACSAria III flow cytometer.

### Statistics

The image was analyzed by QuPath software [17]. The flow cytometry data were analyzed by FlowJo V10.6.2. Statistical significance was determined using a two-tailed unpaired Student’s *t*-test, with significance reported as *p* values < 0.05. * represents *p* ≤ 0.05, ** represents *p* ≤ 0.01, and *** represents *p* ≤ 0.001. All calculations and quantitative plots were performed using GraphPad Prism 8.0 (GraphPad Software Inc.).

## Results

### The spatial distribution of cholesterol and APOE in tumor-bearing brain

**Fig. 1:**
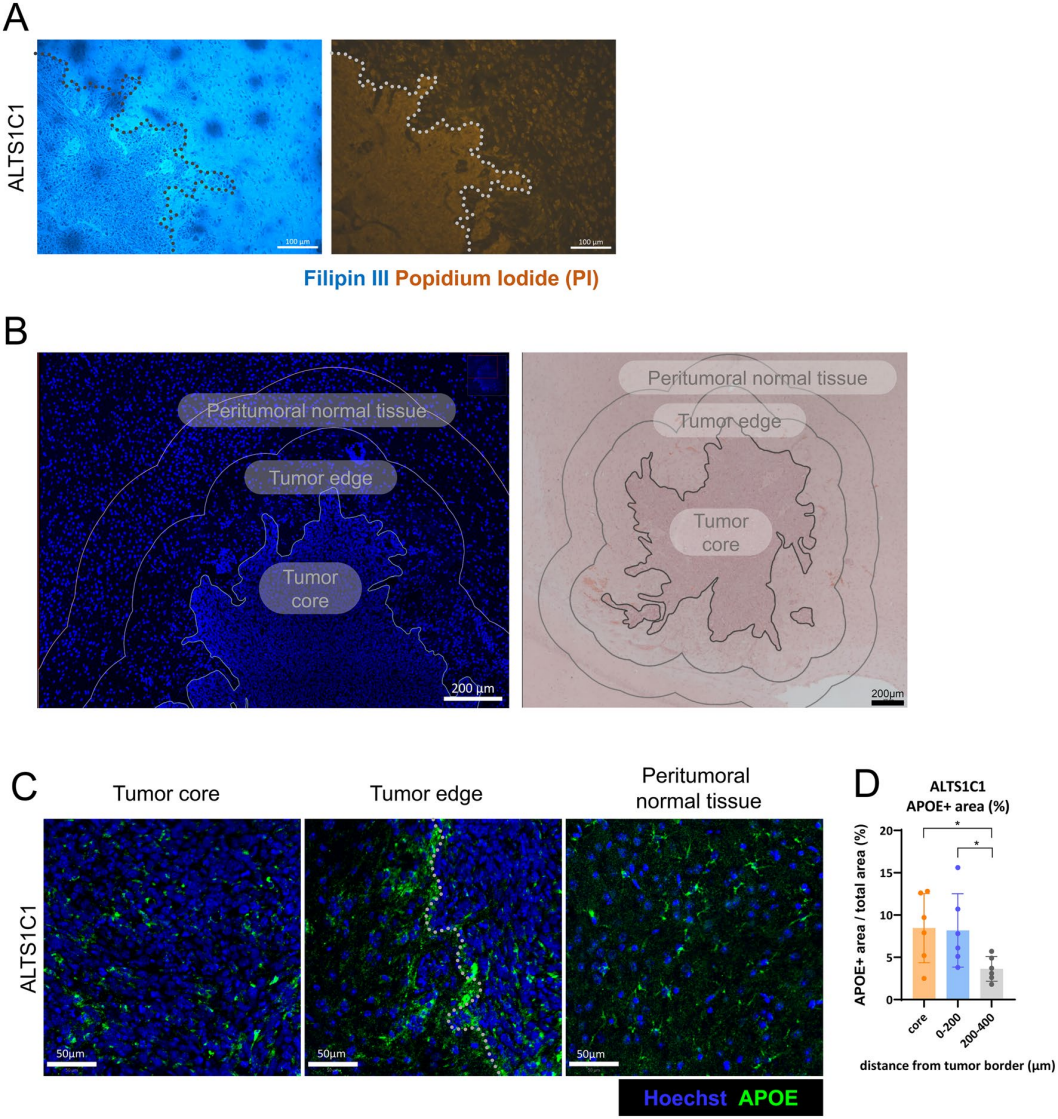
The spatial distribution of cholesterol and APOE in ALTS1C1 tumor-bearing brain. **A** Representative images of Filipin III staining (blue) on day 18 brain tumor tissue. The tumor area was recognized by the higher nucleus (Propidium Iodide stain, orange) density. Scale bar = 100 μm. **B** Tissue segmentation for spatial analysis with immunofluorescent staining and H&E staining. H&E image revealed that the ALTS1C1 tumor exhibited an irregular and infiltrative pattern. **C** Representative images of APOE (green) immunofluorescence (IF) staining. The nucleus was detected with Hoechst staining (blue). Scale bar = 50 μm. **D** Quantification of APOE IF staining in different regions (n = 6). Data are presented as mean ± SD. Statistical significance was determined using a two-tailed unpaired Student’s *t*-test. * represents *p* ≤ 0.05, ** represents *p* ≤ 0.01, and *** represents *p* ≤ 0.001

To understand the role of cholesterol in brain tumor invasion, this study examined the ALTS1C1 murine astrocytoma model. Cholesterol level in the brain TME was first assessed with Filipin III staining on tissue sections from mice bearing ALTS1C1. The mice were intracranially injected with tumor cells and killed 18 days after inoculation. Results showed a higher Filipin signal in the tumor-surrounding tissue, suggesting localized cholesterol accumulation (Fig. 1A). To examine the spatial distribution of APOE, the tumor-containing brain was divided into three parts using QuPath software based on Hoechst and H&E staining: the tumor core, the tumor edge, defined as the area extending 200 µm from the tumor border, and the peritumoral normal tissue, defined as the region extending 200–400 µm from the tumor border (Fig. 1B). Immunofluorescence (IF) staining showed a distinct APOE signal at the tumor border (Fig. 1C). The percentage of APOE-positive areas in these regions was quantified using QuPath software. The percentage of APOE in the tumor core (8.5 ± 4.1%) and tumor edge (8.2 ± 4.3%) was significantly higher than that in the peritumoral normal tissue (3.6 ± 1.5%) in the ALTS1C1 model (Fig. 1D). Cholesterol was also seen to be accumulated in the tumor edge of GL261 murine glioblastoma model (Supplementary Fig. 1A). Similarly, the GL261 model showed more APOE signal in tumor edge (12.0 ± 9.5%) than peritumoral normal tissue (4.0 ± 3.3%). In addition, the APOE signal was slightly higher in the tumor edge than in the tumor core in the GL261 model (Supplementary Fig. 1B, C, D). The above findings demonstrate that cholesterol and APOE are primarily localized in the tumor-surrounding tissue.

### GFAP and APOE were elevated in the tumor edge

**Fig. 2:**
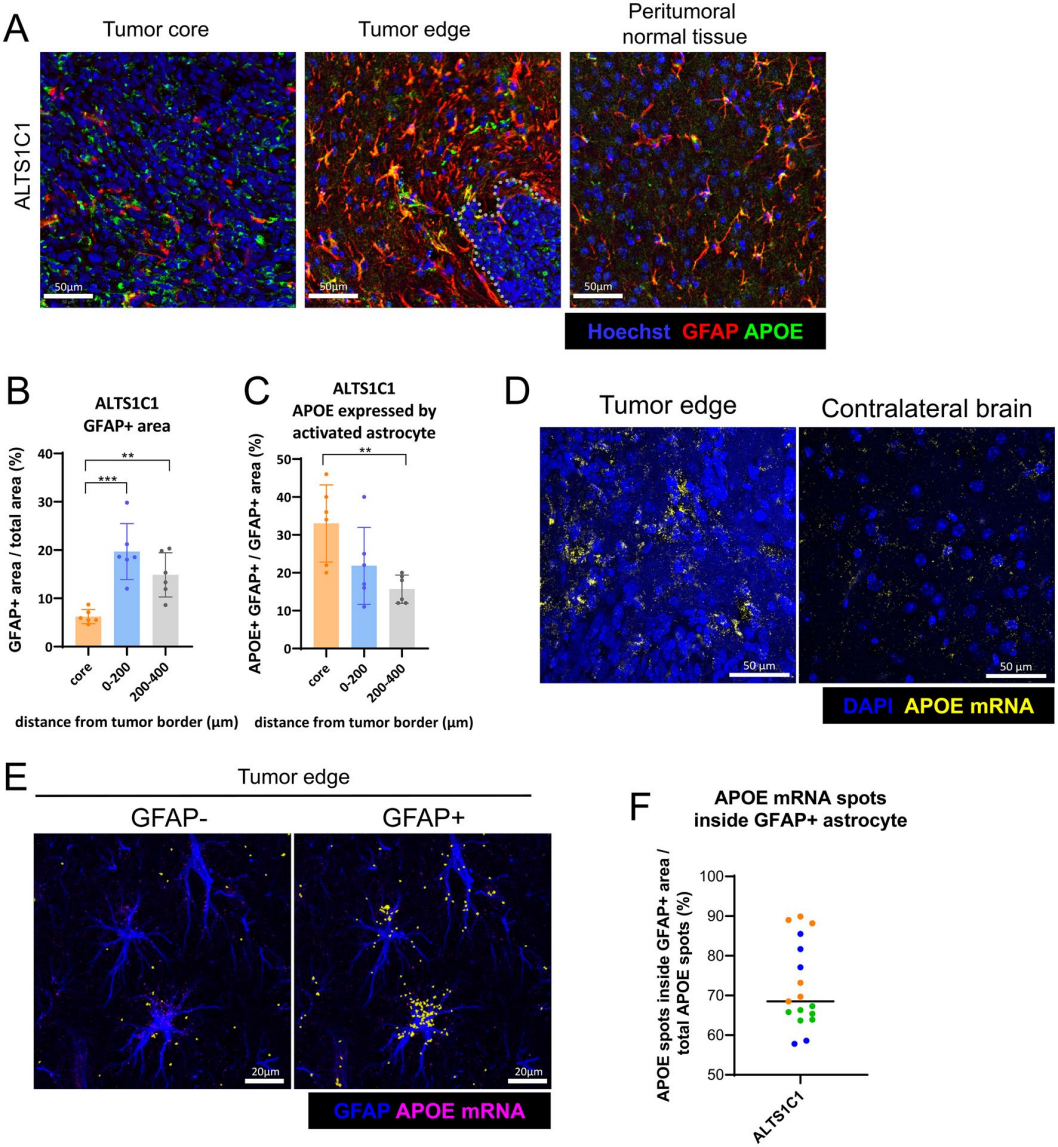
APOE and GFAP expression of astrocytes were analyzed with IF staining and IF-ISH dual staining. **A** Representative IF images of GFAP (red) and APOE (green) co-staining. The nucleus was detected with Hoechst staining (blue). Scale bar = 50 μm. **B** Quantification of the percentage of GFAP + area from IF images (n = 6). **C** Quantification of the percentage of APOE expressed by GFAP + activated astrocyte from IF images (n = 6). Data are presented as mean ± SD. **D** Representative images of ISH staining showing APOE mRNA (yellow). Nuclei were stained with DAPI (blue). Scale bar = 50 μm. **E** Representative images of ISH and IF dual staining. Astrocytes were labeled with GFAP IF staining (blue) and APOE mRNA was detected using ISH (magenta). Scale bar = 20 μm.** F** Quantification of APOE mRNA spots located in GFAP − and GFAP + regions, identified using QuPath software and marked in yellow as shown in Fig. 3E. Data were quantified from three mice per tumor model, with 5–6 randomly selected images per tumor section. Data points from the same section are color-coded (orange, blue, and green), with each point representing a single image. Data are presented as mean. Statistical significance was determined using a two-tailed unpaired Student’s *t*-test. * represents *p* ≤ 0.05, ** represents *p* ≤ 0.01, and *** represents *p* ≤ 0.001

To further explore the relationship between the increased APOE production and astrocytes, IF double staining for GFAP and APOE was performed (Fig. 2A). The expression levels of APOE and GFAP in different regions were quantified as described in Fig. 1B. Clear accumulation of GFAP at the tumor edge was observed in the ALTS1C1 tumor (19.7 ± 5.8% at the edge compared to 6.2 ± 1.5% and 14.9 ± 4.6% at tumor core and peritumoral normal tissue, respectively) (Fig. 2B). Since ALTS1C1 was originally derived from primary astrocytes, to further exclude the possibility that the GFAP signal might come from ALTS1C1, GFAP IF staining on the ALTS1C1-GFP brain tumor section was performed. The results showed that most of the ALTS1C1-GFP cells expressed low or no GFAP signal, which may not interfere with GFAP quantification (Supplementary Fig. 2). To examine whether activated astrocytes express higher APOE in the tumor edge, APOE expressed by activated astrocytes was quantified as APOE + GFAP + area. The APOE + GFAP + area was divided by total GFAP + area to examine the percentage of APOE expressing activated astrocytes in total activated astrocytes. The APOE expressing activated astrocyte was increased at the tumor edge (21.8 ± 10.2%) and significantly increased at the tumor core (33.0 ± 10.2%) compared to the peritumoral normal tissue (15.7 ± 3.7%) (Fig. 2C). A similar increase in activated astrocyte and APOE expressing activated astrocyte at the tumor edge was also found in the GL261 model (Supplementary Fig. 3A–C).

### APOE is mainly expressed by activated astrocytes at the tumor edge

To further validate the increased APOE production by astrocytes, the expression of APOE mRNA was examined using the ViewRNA ISH kit. An increased number of APOE mRNA spots (APOE spots) at the tumor edge, compared with the contralateral brain, was observed (Fig. 2D). Furthermore, GFAP IF staining was combined with APOE ISH staining to confirm APOE secretion from activated astrocytes (Fig. 2E). The APOE spots located inside and outside the GFAP + area were quantified using QuPath software. The results showed that the majority of APOE spots were found within GFAP + astrocytes at the tumor edge in the ALTS1C1 model (72.5 ± 10.7%) (Fig. 2F). Similar findings were also found in GL261 model where 74.5 ± 8.9% of APOE spots come from GFAP + astrocyte (Supplementary Fig. 3D–F). These findings confirm the increased APOE secretion at the tumor edge and demonstrate that the accumulated APOE protein in the tumor edge is primarily produced by astrocytes.

### Macrophages are associated with the alteration of APOE within the tumor

**Fig. 3:**
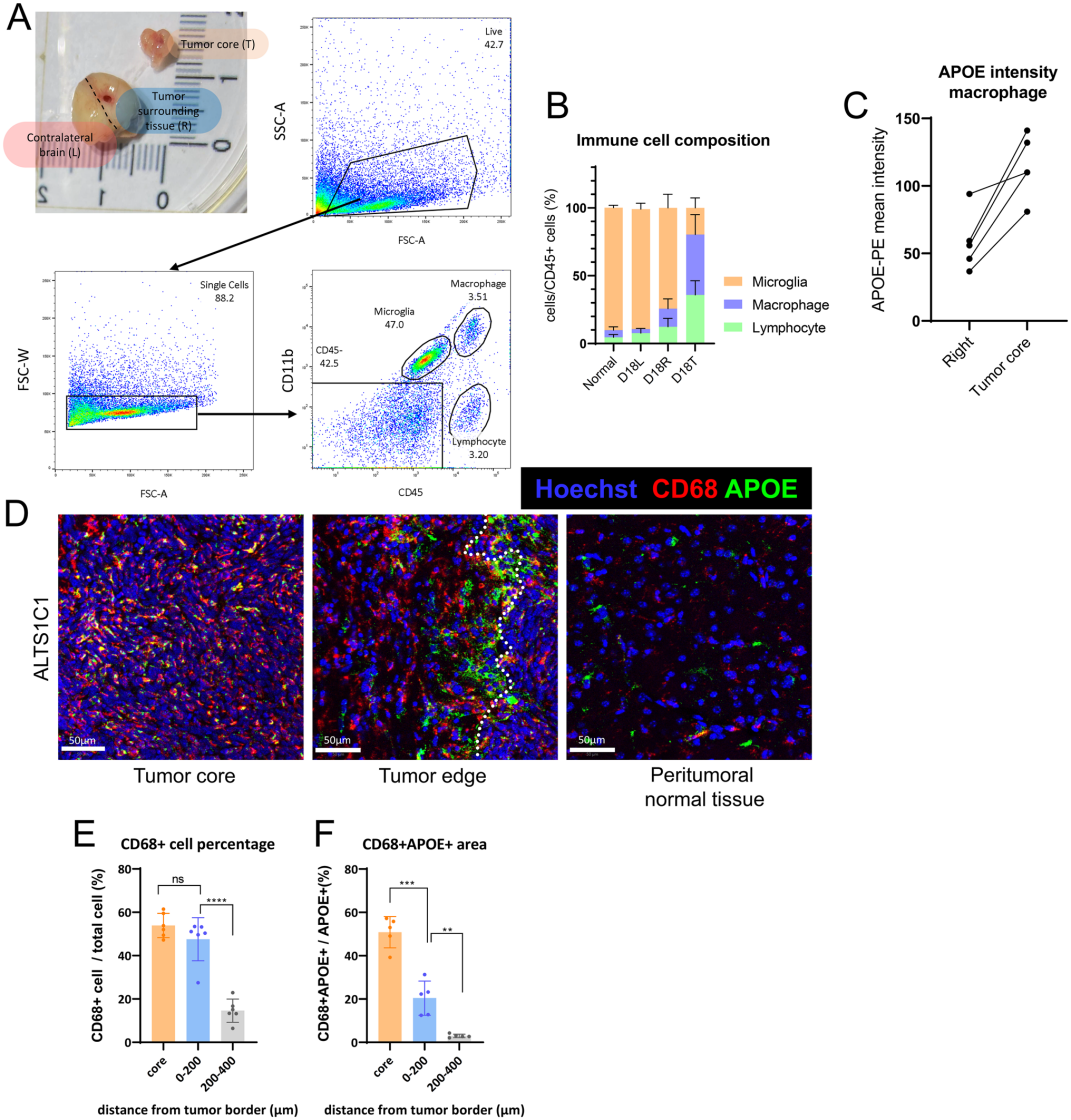
Flow cytometry analysis and IF staining of macrophage in ALTS1C1 model. **A** Representative images of tumor dissection method used during tissue dissociation process and the gating strategy for flow cytometry analysis. The tumor and the brain tissue were collected 18 days after tumor cell inoculation. “L, R, T” indicate the left hemisphere (contralateral brain), right hemisphere (tumor-surrounding tissue), and tumor core, respectively. Microglia were gated as CD45low, CD11b + cells. Macrophages were gated as CD45 high, CD11b + cells. Lymphocytes were gated as CD45 +, CD11b- cells. **B** Immune cell composition in different areas (n = 6). **C** APOE intensity of CD68+ macrophage in right hemisphere versus tumor core tissue (n = 5). **D** Representative images of CD68 (red) and APOE (green) IF co-staining. The nucleus was detected with Hoechst (blue). Scale bar = 50 μm. **E** Quantification of CD68+ cells from the IF images (n = 6). **F** Quantification of CD68+ APOE+ cells from the IF images (n = 5). Data are presented as mean ± SD. Statistical significance was determined using a two-tailed unpaired Student’s *t*-test. * represents *p* ≤ 0.05, ** represents *p* ≤ 0.01, and *** represents *p* ≤ 0.001

The above results have demonstrated higher APOE levels in both the tumor core and tumor edge, with astrocytes being the main contributors at the tumor edge. However, there was also increased APOE expression in the tumor core. Flow cytometry analysis of the cell composition in the ALTS1C1 tumor was performed to further identify the source of APOE in the tumor core. The day 18 tumor-containing brain tissue was dissected into three parts: tumor core tissue (T), right hemisphere without tumor tissue (R), and left ‘normal brain’ hemisphere (L). The right hemisphere (R) was considered the tumor-surrounding tissue that includes the tumor edge and peri-tumor normal tissues described in Fig. 1B for IF analysis. The brains of age-matched healthy mice were used as normal brain tissue for comparison. Each cell population was identified based on the expression of CD45 and CD11b (Fig. 3A). Resident microglia were the most abundant immune cells in normal brain tissue (90.2 ± 1.8%) (Fig. 3B). In day 18 tumor-surrounding tissue (D18R), the percentages of lymphocytes (4.6 ± 2% to 12.4 ± 6.2%) and macrophages (5.3 ± 2.5% to 13.3 ± 7.3%) increased compared to normal brain tissue. Macrophages became the dominant cell type (44.4 ± 14.7%) in day 18 tumor core tissue (D18T) (Fig. 3B). In addition, flow cytometry analysis showed that CD45 high macrophages expressed higher levels of APOE in the tumor core than in the surrounding tissue across all five tumors on day 18 (Fig. 3C).

Consequently, the expression of APOE by macrophages was analyzed through IF staining (Fig. 3D). Quantification of CD68 signals showed that macrophages constituted approximately half of the cells in the tumor core tissue (53.9 ± 5.6%), with the percentage slightly decreasing in the tumor edge (47.5 ± 10.0%) (Fig. 3E), which is consistent with the flow cytometry analysis. The proportion of APOE and CD68 double-positive areas was further quantified to identify the source of APOE. The double-positive area was increased at the tumor edge (20.4 ± 7.9%) and significantly increased in the tumor core (50.9 ± 7.2%) compared to peritumoral normal tissue (3.0 ± 0.8%) in the ALTS1C1 model (Fig. 3F). In addition, ISH staining of APOE mRNA revealed that the RNA spots were sparsely distributed within tumor core (Supplementary Fig. 4), with strong signal accumulation in a few scattered cells, indicating highly localized expression. However, due to technical limitations, APOE ISH staining cannot be co-performed with CD68 IF staining. The APOE secretion by macrophages could not be directly confirmed within tumor core.

The higher percentage of CD68 and APOE double-positive areas in the tumor core compared to the tumor edge and peritumoral normal tissue indicates that macrophages are also associated with the increased APOE in the tumor microenvironment, especially within the tumor core.

## Discussion

Despite the important role of astrocytes in supporting brain homeostasis, their role in brain TME has been less studied. Here, we provide evidence about how astrocytes could contribute to brain TME. Our IF staining results showed a higher amount of cholesterol and APOE at the tumor edge than at the tumor core. These findings are consistent with previous research in the GL261 mouse brain tumor model, showing a higher presence of APOE at the tumor edge [6, 12]. Still, it was unclear which cells were responsible for the increased APOE. This study used ViewRNA ISH staining to investigate the expression of APOE at the RNA level. This method enables the visualization of each mRNA molecule within the cell, with each spot representing a single mRNA molecule, and combining IF staining with ViewRNA ISH staining helps to identify cells responsible for the increased APOE. This study is the first demonstration that activated astrocytes with strong GFAP signals were the primary cells responsible for the APOE production at the brain tumor edge.

The observation that both APOE and cholesterol accumulate primarily in the tumor-surrounding tissue is consistent with our previous finding that the tumor edge exhibits unique characteristics, including higher microvascular density (MVD) and better vessel function, but expresses more CAIX [9, 16]. The CAIX, which is highly expressed by cells under anaerobic metabolism and frequently used as a hypoxia marker, was increasingly expressed in the tumor edge area. In addition, CAIX expression showed good spatial correlation with the astrocyte marker GFAP, indicating excessive carbon dioxide production by peripheral astrocytes. Consistent with our earlier work on active astrocytes at the tumor edge, the accumulation of APOE in this region may reflect the altered metabolic state of reactive astrocytes. Although the regulatory mechanism underlying the upregulation of APOE in astrocytes was not explored in this study, emerging evidence suggests that APOE expression in astrocytes may be influenced by various microenvironmental factors. Previous research revealed that the cholesterol biosynthesis pathway and APOE were upregulated in hypoxia-exposed primary astrocytes [18, 19]. It has been suggested that TAAs become activated within the tumor microenvironment in the presence of tumor cells and microglia [7]. In addition, another study found that GBM co-culture or treatment with a GBM-conditioned medium induces the expression of rate-limiting enzymes involved in de novo cholesterol synthesis within astrocytes [6]. It was also found that treatment with IL-1β and Epidermal growth factor (EGF) increases APOE secretion in astrocytes [20, 21]. In this study, the proximity of astrocytes to macrophages and tumor cells at the tumor edge further supports the notion that cellular crosstalk between these components may play a crucial role.

In this study, we have shown that APOE secreted by astrocytes accumulated at the tumor edge. This observation may suggest potential roles for astrocyte-derived APOE in supporting glioma progression. This can be explained from three different perspectives: (1) indirect effect through lipid transport, (2) indirect effect via immune modulation, and (3) direct effect on tumor cell proliferation and migration. First, astrocytes could contribute to lipid delivery by secreting APOE, which facilitates lipid transport to tumor cells. The proximity of astrocytes to the hypoxic region suggests that these cells may help to meet the metabolic needs of the tumor, possibly by providing lipids or other essential metabolites. For example, a study reported that lipid droplets accumulate in the hypoxic areas of GBM organoids, and that oxygen availability plays a role in controlling lipid metabolism and maintaining stem-like properties [23, 24]. Another study reported that knockdown of astrocytic ATP-binding cassette transporter A1 (ABCA1), which is responsible for cholesterol trafficking, with the injection of Gfap-shAbca1 into GL261-bearing mice led to decreased tumor cholesterol and induction of apoptotic cell death [6]. Second, APOE may also contribute to immune regulation. One study found that knocking down APOE in glioblastoma cells altered cytokine secretion (increased IL-6/IL-12/TNF-α and decreased CCL5/TGF-β) and reduced the proportion of M2 macrophages in a co-culture system [22]. Another study showed that activation of the LXR/ApoE axis in murine models reduced myeloid-derived suppressor cell (MDSC) populations, promoted cytotoxic T cell responses, and enhanced the efficacy of immunotherapies across multiple cancers [23], suggesting the immunomodulatory function of APOE. Third, APOE may also contribute to the regulation of TME beyond lipid metabolism. A previous study showed that APOE knockdown in U87 and U251 glioma cells reduced cell viability, migration, and invasion [22]. Other studies in non-CNS cancer models also support this possibility. For instance, in colorectal cancer, APOE enhances tumor cell migration and invasion through the Jun-APOE-LRP1 signaling axis [24]. In ovarian cancer, APOE was shown to be essential for cell survival and proliferation. Silencing APOE induced G2 arrest and apoptosis [25]. Together, these studies suggest the versatile role of APOE in tumor progression, including lipid metabolism, immune regulation, and tumor cell intrinsic effects.

Previous research has reported that the cholesterol-rich TME caused the increased cholesterol levels of TAMs in the mouse glioblastoma model, impairing their phagocytosis ability [12]. Conversely, another study reported that cancer cells can scavenge the cholesterol from the microenvironment by upregulating apolipoprotein and its receptor in mouse ovarian cancer. This may lead to cholesterol depletion in tumor stromal cells, including macrophages. Increased macrophage cholesterol efflux then leads to the anti-inflammatory phenotype [26]. The above two contradictory findings regarding the role of apolipoprotein in regulating macrophage function in two different tumor cell lines highlight the importance of the TME in shaping the immune profile. As reported in our previous research, TAMs show phenotypic changes across different regions in brain TME. Most TAMs in the tumor core are double-positive for CD68 and F4/80, but the TAMs at the tumor edge are more F4/80+ than CD68+ [16]. In this study, APOE was also associated with CD68 in the tumor core but not the tumor edge. Despite the significant increase in APOE in the tumor core astrocytes, their small number suggests that the accumulated APOE seen in the ALTS1C1 tumor core may originate from other cells. The localized pattern of APOE RNA supports the idea that only a subset of cells may be actively expressing APOE in the tumor core. Those APOE may be secreted either by tumor cells or macrophages. The spatially distinct correlation of APOE with both macrophages and astrocytes in brain tumors also suggests the different metabolic microenvironments and regulatory mechanisms within and around the tumor.

In summary, we have shown that astrocytes drive the accumulation of apolipoprotein E at the brain tumor edge. Our findings provide a foundation for future research. Future studies of knocking down the APOE in the astrocyte may help to uncover their role in brain tumor progression. In addition, in vitro studies of co-culturing astrocytes with tumor cells could further clarify the contribution of other cells in the activation of astrocytes. There is still much to learn about the role of astrocytes in the TME. More studies are needed to uncover their contribution and potential as a therapeutic target in glioma treatment.

## Supplementary Information

Below is the link to the electronic supplementary material.

Supplementary file1 (DOCX 169281 KB)

## Data Availability

All data presented within the article and its supplementary information files are available upon request from the corresponding author.

## References

[1] Sofroniew MV, Vinters HV (2010) Astrocytes: biology and pathology. Acta Neuropathol 119(1):7–35 20012068 10.1007/s00401-009-0619-8 PMC2799634

[2] Xu Q et al (2006) Profile and regulation of apolipoprotein E (ApoE) expression in the CNS in mice with targeting of green fluorescent protein gene to the ApoE locus. J Neurosci 26(19):4985–4994 16687490 10.1523/JNEUROSCI.5476-05.2006 PMC6674234

[3] Huang Y, Mahley RW (2014) Apolipoprotein E: structure and function in lipid metabolism, neurobiology, and Alzheimer’s diseases. Neurobiol Dis 72 Pt A:3–124 25173806 10.1016/j.nbd.2014.08.025 PMC4253862

[4] Corraliza-Gomez M, Sanchez D, Ganfornina MD (2019) Lipid-binding proteins in brain health and disease. Front Neurol 10:1152 31787919 10.3389/fneur.2019.01152 PMC6854030

[5] Brandao M et al (2019) Astrocytes, the rising stars of the glioblastoma microenvironment. Glia 67(5):779–790 30240060 10.1002/glia.23520

[6] Perelroizen R et al (2022) Astrocyte immunometabolic regulation of the tumour microenvironment drives glioblastoma pathogenicity. Brain 145(9):3288–3307 35899587 10.1093/brain/awac222 PMC10233310

[7] Henrik Heiland D et al (2019) Tumor-associated reactive astrocytes aid the evolution of immunosuppressive environment in glioblastoma. Nat Commun 10(1):2541 31186414 10.1038/s41467-019-10493-6 PMC6559986

[8] Li H et al (2024) Tumor-associated astrocytes promote tumor progression of Sonic Hedgehog medulloblastoma by secreting lipocalin-2. Brain Pathol 34(1):e13212 37721122 10.1111/bpa.13212 PMC10711256

[9] Lin CM et al (2019) Distinct tumor microenvironment at tumor edge as a result of astrocyte activation is associated with therapeutic resistance for brain tumor. Front Oncol 9:307 31106146 10.3389/fonc.2019.00307 PMC6498880

[10] Villa GR et al (2016) An LXR-cholesterol axis creates a metabolic co-dependency for brain cancers. Cancer Cell 30(5):683–693 27746144 10.1016/j.ccell.2016.09.008 PMC5479636

[11] Gu D et al (2023) Sterol regulatory element-binding protein 2 maintains glioblastoma stem cells by keeping the balance between cholesterol biosynthesis and uptake. Neuro Oncol 25(9):1578–1591 36934350 10.1093/neuonc/noad060 PMC10651206

[12] Wang S et al (2023) Oncolytic viruses engineered to enforce cholesterol efflux restore tumor-associated macrophage phagocytosis and anti-tumor immunity in glioblastoma. Nat Commun 14(1):4367 37474548 10.1038/s41467-023-39683-z PMC10359270

[13] Comba A et al (2021) Uncovering spatiotemporal heterogeneity of high-grade gliomas: from disease biology to therapeutic implications. Front Oncol 11:703764 34422657 10.3389/fonc.2021.703764 PMC8377724

[14] Garcia-Diaz C et al (2023) Glioblastoma cell fate is differentially regulated by the microenvironments of the tumor bulk and infiltrative margin. Cell Rep 42(5):112472 37149862 10.1016/j.celrep.2023.112472

[15] Caponegro MD et al (2021) A distinct microglial subset at the tumor-stroma interface of glioma. Glia 69(7):1767–1781 33704822 10.1002/glia.23991 PMC8113099

[16] Wang SC et al (2012) Tumor-secreted SDF-1 promotes glioma invasiveness and TAM tropism toward hypoxia in a murine astrocytoma model. Lab Invest 92(1):151–162 21894147 10.1038/labinvest.2011.128

[17] Bankhead P et al (2017) QuPath: open source software for digital pathology image analysis. Sci Rep 7(1):16878 29203879 10.1038/s41598-017-17204-5 PMC5715110

[18] Hongyan C et al (2023) The coupling of neuron-astrocyte lipid metabolism induced by neonatal hypoxic-ischaemic brain damage is ApoE dependent. Research Square Platform LLC

[19] Shrivastava V et al (2024) Glial cholesterol redistribution in hypoxic injury in vitro influences oligodendrocyte maturation and myelination. Biochim Biophys Acta Mol Basis Dis 1870(8):167476 39181517 10.1016/j.bbadis.2024.167476

[20] Baskin F et al (1997) Altered apolipoprotein E secretion in cytokine treated human astrocyte cultures. J Neurol Sci 148(1):15–18 9125385 10.1016/s0022-510x(96)05335-x

[21] Aleong R, Blain JF, Poirier J (2008) Pro-inflammatory cytokines modulate glial apolipoprotein E secretion. Curr Alzheimer Res 5(1):33–37 18288929 10.2174/156720508783884666

[22] Huang W et al (2025) APOE drives glioma progression by modulating CCL5/CCR5 signaling in the tumor microenvironment and inducing M2 macrophage polarization. Immunobiology 230(3):152895 40203505 10.1016/j.imbio.2025.152895

[23] Tavazoie MF et al (2018) LXR/ApoE activation restricts innate immune suppression in cancer. Cell 172(4):825-840e18 29336888 10.1016/j.cell.2017.12.026 PMC5846344

[24] He L et al (2023) Jun-APOE-LRP1 axis promotes tumor metastasis in colorectal cancer. Biomol Biomed 23(6):1026–1037 37310025 10.17305/bb.2023.9248 PMC10655886

[25] Chen YC et al (2005) Apolipoprotein E is required for cell proliferation and survival in ovarian cancer. Cancer Res 65(1):331–337 15665311

[26] Goossens P et al (2019) Membrane cholesterol efflux drives tumor-associated macrophage reprogramming and tumor progression. Cell Metab 29(6):1376-1389e4 30930171 10.1016/j.cmet.2019.02.016

