# Supplementary material for: Activated astrocytes drive the accumulation of apolipoprotein E at the brain tumor edge
Source: Brain Tumor Pathol. 2025 Jul 31;43(1):18–28. doi: 10.1007/s10014-025-00511-5 (PMC12819506; doi:10.1007/s10014-025-00511-5)
Supplement: Supplementary file 1 — Supplementary file1 (DOCX 169281 KB) [file 10014_2025_511_MOESM1_ESM.docx]

**Supplemental Figure 1**

**The spatial distribution of Cholesterol and APOE in tumor-bearing brain** (A) Representative images of Filipin III staining (blue) on day18 brain tumor tissue. The tumor area was recognized by the higher nucleus (Propidium Iodide stain, orange) density. Scale bar = 200 μm. (B) Illustration of tissue segmentation for spatial analysis with immunofluorescent staining and H&E staining. H&E image revealed that the GL261 tumor showed a more compact shape with internal cavities compared with the ALTS1C1 tumor. (C) Representative images of APOE (green) immunofluorescence (IF) staining. The nucleus was detected with Hoechst staining (blue). Scale bar = 50 μm. (D) Quantification of APOE IF staining in different regions (n=3). Data are presented as mean ± SD. Statistical significance was determined using a two-tailed unpaired Student’s t-test. * represents p ≤ 0.05, ** represents p ≤ 0.01, and *** represents p ≤ 0.001.

**Supplemental Figure 2**

**GFAP IF staining on the ALTS1C1-GFP brain tumor section.** Representative IF images of GFAP (red) on the ALTS1C1-GFP (green) day18 brain tumor section. The nucleus was detected with Hoechst staining (blue). Scale bar = 20 μm.

**Supplemental Figure 3**

**APOE and GFAP expression of astrocytes were analyzed with IF staining and IF-ISH dual staining.** (A) Representative IF images of GFAP (red) and APOE (green) co-staining. The nucleus was detected with Hoechst staining (blue). Scale bar = 50 μm. (B) Quantification of the percentage of GFAP+ area from IF images (n=3). (C) Quantification of the percentage of APOE expressed by GFAP+ activated astrocyte from IF images (n=3). Data are presented as mean ± SD. (D) Representative images of ISH staining showing APOE mRNA (yellow). Nuclei were stained with DAPI (blue). Scale bar = 20 μm. (E) Representative images of ISH and IF dual staining**.** Astrocytes were labeled with GFAP IF staining (blue) and APOE mRNA was detected using ISH (magenta). Scale bar = 20 μm. **(F**) Quantification of APOE mRNA spots located in GFAP− and GFAP+ regions, identified using QuPath software and marked in yellow as shown in Supplementary Fig. 3E. Data were quantified from three mice per tumor model, with 5 to 6 randomly selected images per tumor section. Data points from the same section are color-coded (orange, blue, and green), with each point representing a single image. Data are presented as mean. Statistical significance was determined using a two-tailed unpaired Student’s t-test. * represents p ≤ 0.05, ** represents p ≤ 0.01, and *** represents p ≤ 0.001.

**Supplemental Figure 4**

**Combining ISH and IF staining of APOE mRNA and GFAP protein in tumor core.** Nuclei were stained with DAPI (gray). Astrocytes were labeled with GFAP IF staining (blue) and APOE mRNA was detected using ISH (yellow). Scale bar = 20 μm.
